# The Biology and Ecology of the Emerald Ash Borer, *Agrilus planipennis*, in China

**DOI:** 10.1673/031.010.12801

**Published:** 2010-08-09

**Authors:** Xiao-Yi Wang, Zhong-Qi Yang, Juli R. Gould, Yi-Nan Zhang, Gui-Jun Liu, EnShan Liu

**Affiliations:** ^1^ The Key Laboratory of Forest Protection, State Forestry Administration, Research Institute of Forest Ecology, Environment and Protection, Chinese Academy of Forestry, Beijing 100091, China; ^2^ Animal and Plant Health Inspection Service, United States Department of Agriculture, 1398 West Truck Road, Buzzards Bay, MA 02542, USA; ^3^ Beijing Vocational College of Agriculture, Beijing 102442 China; ^4^ Guangang Forest Park, Dagang District, Tianjin 300274, China

**Keywords:** Buprestidae, behavior, life cycle, management

## Abstract

The biology, ecology, and life cycle of the emerald ash borer, *Agrilus planipennis* Fairmaire (Coleoptera: Buprestidae), were studied using regular inspection in the forest and observations in the laboratory. Results indicated that *A. planipennis* are mostly univoltine in Tianjin, China. They overwintered individually as mature larvae in shallow chambers excavated in the outer sapwood. In late July, some full-grown larvae began to build overwintering chambers, and all larvae entered the sapwood for dormancy by early November. *A. planipennis* pupated in the overwintering chamber from early April to mid May the following year, and the average pupal duration was about 20 days. In late April, some newly eclosed adults could be found in the pupal cells, but they had not yet emerged from the tree. Adults began to emerge in early May, with peak flight occurring in mid May. The average longevity of adults was about 21 days and the adult stage lasted through early July. The adults fed on ash foliage as a source of nutrition. Mating was usually conducted and completed on the leaf or trunk surfaces of ash trees. Oviposition began in mid May and eggs hatched on average in 15.7 days. The first instar larvae appeared in early June. The larval stage lasted about 300 days to complete an entire generation. The emerald ash borer had four larval instars on velvet ash, *Fraxinus velutina* (Scrophulariales: Oleaceae). The major natural control factors of *A. planipennis* were also investigated, and preliminary suggestions for its integrated management are proposed.

## Introduction

The emerald ash borer, *Agrilus planipennis* Fairmaire (Coleoptera: Buprestidae) (= *A. marcopoli* Obenberger), is an important wood-boring beetle injuring ash trees (*Fraxinus* spp.) (Oleaceae) in Asia. There are 27 species and one sub-species of *Fraxinus* native to China (Wei 1992), with eight species commonly planted in plantations. The emerald ash borer mainly infests *F. mandshurica, F. rhynchophylla* and *F. chinensis* (Hou 1986; Yu 1992), but also attacks *F. pennsylvanica, F. americana* and *F. velutina* (Zhao et al. 2005), which are native to North America but are planted in China. Many species in the genus are important timber resources, especially *F. mandshurica* and *F. rhynchophylla. Fraxinus* trees are often used as ornamental and street trees, as well as wind breaks to protect agriculture in farmlands or coastal regions in China. Ash trees can endure saline-alkaline soil and are especially valuable in salty coastal regions such as Tianjin, China (Wei 1992).

*A. planipennis* larvae feed on the phloem, cambium, and shallow sapwood under ash tree bark. It is very difficult to detect and control *A. planipennis* because of its concealed life history. In the 1960s, *A. planipennis* severely infested *F. americana* in northeastern China (Liu 1966; Hou 1986; Yu 1992). Recently, outbreaks of this pest arose on *F. velutina* in parts of Tianjin, China, threatening the main street and park trees. Because of the severity of this pest, preliminary observations on its habits were conducted (Zhang et al. 1995; Liu et al. 1996).

In June 2002, *A. planipennis* was reported to be seriously infesting white ash (*F. americana*), green ash (*F. pennsylvanica*) and black ash (*F. nigra*) in parts of North America, including Michigan and Ohio in the USA and Ontario in Canada. Subsequently, a large number of ash trees died in those areas (Haack et al. 2002). Because *A. planipennis* is only sometimes an important pest in certain areas in China, information on its biology and ecology is extremely limited. Therefore, the biology of *A. planipennis* was investigated in Tianjin, China from 2003 to 2004 to obtain information about its life cycle and ecological habits, which will contribute to the scientific and effective integrated management of this beetle.

## Materials and Methods

### The research site

This research was conducted in one stand of 9- to 10-year-old velvet ash, *F. velutina* Torr., with diameters at breast height (DBH) of 5–10 cm, in the Guangang Forest Park (38° 56′ N, 117° 29′ E), Dagang District, Tianjin Municipality, China, which is a part of the sea coast protection forests of the city. The annual average temperature in this area is 12.1° C, with maximum temperature reaching 40.3° C, lowest temperature at -20.3° C, and a rainfall of 500–700 mm. Because the forest is near the Bohai seashore, the area has heavy saline-alkaline soil with a high underground water table. The salt content of the soil is approximately 1–1.5%, with the highest recorded alkalinity at 2.47%. Because the velvet ash tolerates salt, this tree has been planted widely in most districts of Tianjin, and many forests are monoculture ash plantings. There are approximately 130 ha of ash trees in the Guangang Forest Park. The selected research site was one 5 ha plot where the trees were planted from east to west with 1.5 m row spacing and 1.0 m spacing between trees.

### Infestation rates and insect density

Twelve ash trees infested with *A. planipennis* were felled at random in the research plot on August 15, 2003 (DBH ranged from 2.86 cm to 8.28 cm). The bark was completely removed, and the following records were taken: DBH (precision 1 mm), the height above the ground where *A. planipennis* larvae occurred, the number of live and dead insects, and observed mortality factors. In addition, from late October to early November 2003, the infestation rates and insect density were surveyed in seven other fields in the Guangang Forest Park. Twenty-five trees (5 trees in each cardinal direction of the field, i.e., the east, south, west, north, and center) were selected at random in every field. Each tree was examined by peeling the trunk bark above ground up to the height of 2.5 m. Most of the boring insects in a tree could be found in this range because the trees were still young. Each tree was categorized as having smooth or rough bark. Then, the DBH was measured with a leather measuring tape (precision 1 mm); the number of *A. planipennis* larvae was counted, and the length of *A. planipennis* larvae was measured with a stainless steel ruler (precision 0.5 mm). The number of parasitized larvae, mortality caused by other factors (including rainwater, woodpeckers, pathogens, and plant resistance, e.g. *A. planipennis* larvae killed by rapid growth of tree cambium over the gallery), and the direction and height above the ground of *A. planipennis* larvae and exit holes were noted. The infestation rate, i.e. the percentage of infested trees, was then calculated. The damage symptoms of *A. planipennis* larvae, overwintering, and dormancy behaviors were also observed and recorded.

### Investigation of *A. planipennis* life cycle

The life cycle of *A. planipennis* was recorded from August 2003 through August 2004. Observations were made once every other day from 03 August to 06 November 2003. On each sampling occasion, 5–10 trees were sampled at random in the research plot, and 3–6 locations (10 × 30 cm) on the trunk between 0.5 and 1.5 m were debarked. The developmental stages of *A. planipennis* were recorded. On each sampling occasion, at least 20 insects were observed. From November 2003 to March 2004, observations were made once per month because the fourth instar larvae were dormant in the overwintering chambers. From April to August 2004 observations were again made once every other day. On each sampling occasion, the DBH, the number of *A. planipennis* larvae, the length of *A. planipennis* larvae, the number of parasitized larvae, mortality from other factors, and the direction and height above the ground of each *A. planipennis* larva and exit hole were recorded. When the *A. planipennis* became adults, they were observed every other day in the forest. The temperature and relative humidity were recorded for the periods when observations were made using a portable hygrothermograph. Other weather conditions such as rainfall, cloud cover, or wind were also recorded during the investigations. Data on the natural enemies of *A. planipennis* were collected through the observations in the forest or culture results in the laboratory during the period of *A. planipennis* life cycle survey.

### Determination of overwintering state of *A. planipennis* larvae

To determine whether *A. planipennis* larvae are dormant or in diapauses during the winter, several groups of treatments were observed or conducted both in the laboratory and in the field: A) Thirty-five overwintering larvae of *A. planipennis* were collected in November 2003 from Tianjin, and they were reared in ash branch segments with 1–1.5 cm in diameter and 5–10 cm in length, sealed with wax at the two ends, under room conditions (about 18–22° C). The *A. planipennis* developmental stage was checked and recorded every week, including the numbers of insects that pupated or emerged. B) Two *A. planipennis* infested ash trees with DBH around 12 cm were felled in mid September 2005 from Qinhuangdao City of Hebei Province, and then they were cut into log sections about 50 cm in length, sealed with wax at the two ends, and caged in the laboratory under room conditions (about 18–22° C). The number of *A. planipennis* adults emerging from logs was checked and recorded every week. All the logs were dissected at the end of the experiment. C) A total of 16 overwintering mature *A. planipennis* larvae were put on an artificial diet (one larva per cup) in a growth chamber at 25 ± 1° C and 60–85% RH in January 2005. The number of *A. planipennis* that pupated or emerged was recorded every day. D) (= control) Thirty overwintering *A. planipennis* larvae were collected in November 2003 from Tianjin and reared in ash branch segments with 1–1.5 cm in diameter and 5–10 cm in length as described in treatment A. Segments with insects were stored in the refrigerator at 6 ± 1° C for 65 days and then moved to room conditions. The number of *A. planipennis* that pupated or emerged was recorded every week. E) (= field evaluation) The natural developmental stage of *A. planipennis* was surveyed in the field every other day from early April to mid May 2004.

### Duration of the pupal stage

Overwintering mature larvae (more than 70 individuals) were collected from the forest in early March 2004 and cultured individually in test tubes (diameter 1.5 cm×10 cm) in the laboratory. The degree of development was observed every day, and the dates of pupation and eclosion of each insect were recorded. The temperature and relative humidity were measured both in the laboratory and in the field during the period of observation with portable hygrothermographs.

### Emergence of adults

Thirty trees infested with *A. planipennis* were selected at random in the research plot on 05 May 2004 for the observation of the number of newly emerged adults. The conditions of the trees were recorded, including whether the bark was smooth or rough, DBH, and alive or dead. The numbers, direction, and height of exit holes in every tree were checked and recorded every day. The weather conditions in the field were also recorded. All new exit holes were removed daily using a drawknife to prevent counting the same exit hole twice. Behaviors of adults such as tropism, flight, feeding, mating, and oviposition were also observed and recorded during surveys in the field.

### Determination of adult longevity

*A. planipennis* adults remain in the overwintering chamber for a period of time before emerging from their host tree. Adult longevity, therefore, was observed from two points in time: 1) from the time that the pupae molt to the adult stage (eclosion) and 2) from the time that the adults emerge from the tree. To measure longevity from eclosion to death, newly eclosed adults from pupae cultured in laboratory were placed in a 15 cm diameter Petri dishes (one male and one female per dish). A piece of wet filter paper was put into the dish to maintain moisture, and fresh ash leaves were added daily as food for the adults. Whether each individual was alive or dead was recorded daily for calculating longevity. 24 females and 22 males were observed. To determine longevity from emergence via the exit hole until death, five infested ash trees were felled and sawed into 1 m long segments before *A. planipennis* adults emerged from pupal cells. These wood segments were piled and covered with fine nylon net. The emerged adults were removed from the net daily. A total of 65 adults were collected on 22 May 2004, the peak emergence period, and were placed into two cages in laboratory (26 × 23 × 23 cm). Fresh ash leaves on branches in water were provided for adult food. The ash branches and water in the bottle were renewed every three days. The number and sex of dead *A. planipennis* adults was recorded every day. The adult sex ratio and mating behavior were also observed and recorded in the laboratory.

### Duration of the egg stage

Freshly deposited *A. planipennis* eggs (ivory-white or jade-green) were collected in the field or laboratory beginning in mid May 2004. The eggs were placed individually into glass test tubes (diameter 1.5 cm × 10 cm). The development of eggs was observed daily, and dates of hatch and temperature in the laboratory were recorded. The developmental durations of *A. planipennis* eggs deposited on different dates and relevant temperatures during the experiments (mid May through early July) were determined under ambient temperature in laboratory. The room temperatures were recorded once at 7:00, 12:00, 18:00, and 22:00 every day in order to estimate the daily average temperature and the mean values of daily average temperature during egg development.

### Reproductive status of adult females

Newly emerged *A. planipennis* female adults were collected along with those used to determine adult longevity and were matched with males. After approximately 10 days the females were killed in 75% ethanol. The specimens were fixed on wax dishes with insect needles and dissected under a binocular microscope (20 multiple magnification); the female reproductive system, developmental status, and the number of eggs contained in the ovarioles were observed. A total of 21 *A. planipennis* female adults were dissected.

### Investigation of the geographical distribution of *A. planipennis* in China

In February and March 2004, surveys were conducted in Shandong, Hebei, Liaoning, Jilin, and Heilongjiang Provinces to investigate the distribution of *A. planipennis,* its host plant species, damage status, and natural enemies. One to three counties were surveyed in every province and more than 20 trees were sampled in each of three forests in every county. In addition, Inner Mongolia and the Xingjiang Uygur Autonomous Region were surveyed later in 2006.

### Data analysis

All data were analyzed with the software SAS, version 9.1.3 (SAS Institute Inc. 2006) for variance analysis by the PROC GLM program and for χ*^2^* analysis by PROC FREQ. The longevities of *A. planipennis* adults and the number of *A. planipennis* adults emerged from different status of trees was compared using analysis of variance, and a Tukey's Studentized Range Test (TSRT) was run for separation of means. Sex ratio of adult beetles and the distribution directions of larvae and exit holes, mortalities of trees with rough bark and smooth bark, and the percent of *A. planipennis* larvae or exit holes distributed in trees at different directions were compared using the χ^2^ method.

## Results and Discussion

### Damage symptoms and infestation rates

**Figure 1.**
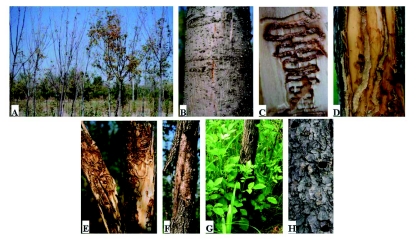
Symptoms of *Agrilus planipennis* infestations: A) Ash forest heavily infested with *A. planipennis*; B) Bark splits on infested trunk; C) Typical larval gallery; D) Vertical galleries; E) High density of *A. planipennis* galleries; F) Bark peeling due to *A. planipennis* infestation; G) Epicormic shoots at base of dead ash tree; H) Adult exit holes. High quality figures are available online.

Generally, weak trees or trees on the edge of forests were most vulnerable to attack by *A. planipennis.* However, *A. planipennis* did attack seemingly healthy trees as well. Young forests with abundant sunlight and low canopy closure were often damaged more seriously (Figure 1A). Infestations of *A. planipennis* were difficult to detect in the early stages of attack. There were rarely any clear symptoms on the surface of the bark during the initial infestation. Sometimes a slight apophysis could be found at the site of infestation, e.g., a vertical slit on smooth bark (Figure 1B). It was more difficult to detect an early stage of infestation for those trees with rough or thick bark.

Newly hatched larvae chewed through the bark and entered into the phloem tissues. They gradually bored into the cambium layer, and then they fed in a zigzag pattern, moving either down or up the trunk. Finally, an S-shaped gallery filled with brown frass was formed between the xylem surface and cambium (Figure 1C). A few larvae bored linearly in a vertical direction, especially when the tree or branch diameter was small (Figure 1D). When densities of *A. planipennis* were high, the trunk was densely covered with galleries, which crossed frequently (Figure 1E), and eventually the bark would fall off (Figure 1F). Infested trees died in 2–3 years once they were girdled by the serpentine galleries because the transportation of nutrients was disrupted. Many epicormic shoots arose from the root or margin of live and dead tissue on the trunk after trees died or were dying (Figure 1G).

When some species of trees in China are attacked by buprestids in the genus *Agrilus,* such as *A. sorocinus, A. mali, A. zanthoxylumi, A. auriventris, A. ratundicollis,* they respond by producing sap at the site of the wound. This makes the site of attack easy to detect. Ash trees, however, do not respond to attack by *A. planipennis* by producing sap, and the only clear evidence of attack is a D-shaped exit hole produced by the emerging adult in the following year (Figure 1H). Therefore populations of *A. planipennis* are only easy to detect when populations of the beetle are quite high and the ash trees are dying.

Based on the survey in August 2003, the infestation rates of velvet ash trees at the Guangang Forest Park, Tianjin, by *A. planipennis* were up to 94.7% in the most severely attacked fields. The infestation of trees with smooth bark was 80.5%, while almost 100% of rough bark trees were infested. Only those trees with very smooth bark, young trees of DBH less than 3 cm, or new shoots were seldom infested by *A. planipennis.* The insect was distributed from the ground to 3.7 m on the main trunk, with the peak density occurring between 0–1.8 m above the ground. A few larvae were also found in the largest branches (diameter around 4–5 cm) on some of the older trees.

### Adult biology

**Sex ratio and longevity.** The adult body is copper-colored or golden green with a metallic glint. The front of the thorax and inner femora of the male are densely covered with long silvery-white setae. These characters are more clear when seen from the side of the insect body or when viewed under a microscope, especially the femora of the middle legs. Corresponding setae on the adult female are short and sparse. In addition, the body of female is commonly wider than that of male when observed from the dorsum. A total of 237 adults (117 females and 120 males) were collected in the field from early May to early June 2004. The sex ratio (female:male) was 0.975:1, which was not significantly different than a sex ratio of 1:1 (χ^2^=0.0380, df = 1, p= 0.8455, *n*= 237).

Adults remained in the pupal cells for an average of 8.67 d (range 5–13 d, *n*= 9) after eclosion. The adults lived an average 20.63 days after eclosion when reared with ash leaves in Petri dishes, with females surviving 20.13 days and males surviving 21.18 days at an average temperature of 21.89° C (range 17–25° C) (Table 1). There were no significant differences between the survival rates of males and females (df = 1, 44, *F* = 0.16, p= 0.6907). The average longevity after emergence from the pupal cell was 21.70 days when the adults were reared in groups in a large cage, with females living 20.56 days and males surviving 22.76 days at an average temperature of 24.05° C (range 21–26.3° C) (Table 1). The longevities show no significant differences between males and females (df = 1, 64, *F* = 0.48, p = 0.49). Adults, therefore, lived an estimated 30.4 d (days in pupal cell plus longevity after exiting the log) when reared in larger cages, compared to only 20.6 days when reared in Petri dishes.

**Figure 2.**
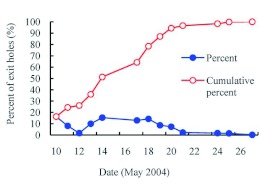
The timing of *Agrilus planipennis* adult emergence in 2004. High quality figures are available online.

**Figure 3.**
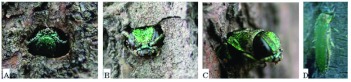
Emergence of *Agrilus planipennis* adult: A) Chewing an exit hole; B & C) The emerging adult; D) Newly emerged adult. High quality figures are available online.

**Emergence.** Field observation of adult emergence from 30 monitored ash trees in 2004 showed that the adult emergence period lasted from early May to the end of May, with the peak in mid May (Figure 2). The adults were most likely to emerge on sunny days when the air temperature was high. Adults could be observed emerging from their pupal chambers between 09:00 and 16:00. Before emergence, a D-shaped exit hole of 3.63 ± 0.071 mm in length (range 3.0–4.5 mm, *n* = 31), 2.77 ± 0.055 mm in width (range 2.0–3.0 mm, *n* = 31) was chewed in the bark (Figure 3A). The adults swallowed the wood chips and excreted frass during emergence, so the pupal cells were packed with frass after adult emergence. The adults alternated between chewing and resting during the emergence process. On an average, it took 25.7 min (range 15–40 min) from the appearance of the vertex of the adult to complete emergence (Figures 3B, 3C). Some adults crawled upwards slowly along the trunk after emergence (Figure 3D), sometimes pausing midway, until they reached the canopy. Other adults flew when they crawled in sunlight and stopped on the leaves of neighboring trees.

The first adult flying in the field was observed on 05 May, while the first exit hole on the observation trees was found on 10 May in 2004. The greatest number of adults was observed emerging on the first day (10 May), which was possibly related to the weather conditions during adult emergence. It was sunny and warm on 04–08 May, but became cloudy and cold on May 9, with lower air temperature and higher relative humidity. Thus the adults stayed in the pupal cells until air temperature warmed on 10 May. Similarly, only a few or no adults emerged when the weather was overcast and rainy on 12, 15 and 16 May,.

**Table 1.**
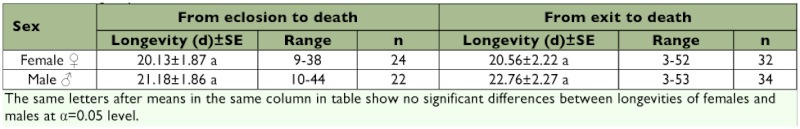
Longevity of the emerald ash borer adults

**Figure 4.**
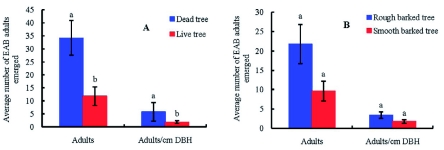
The average number of *Agrilus planipennis* adults emerging from the 30 observed sample trees: A) *A. planipennis* adults emerging from dead trees vs. live trees; B) *A. planipennis* adults emerging from trees with rough bark vs. smooth bark. The different letters on top of bars in the same group show significant differences between the different status of trees at the α = 0.05 level. High quality figures are available online.

The total number of *A. planipennis* adults that emerged from a tree was related to the status or DBH of the tree (Figure 4). An average of 34.2 ± 6.6 adults emerged from the dead trees, which was significantly more than the 11.7 ± 3.5 that emerged from live trees (df = 1, 28, *F* = 10.12, p = 0.004). If the number of adults emerging is converted to the number per diameter at breast height (cm), 5.7 ± 1.0 adults/cm emerged from dead trees, and 1.8 ± 0.5 adults/cm emerged from live trees, which is also significantly different (df = 1, 28, *F* = 13.70, p = 0.0009) (Figure 4A). In addition, an average of 21.8 ± 5.0 adults emerged from the rough barked trees, and 9.6 ± 2.5 emerged from the smooth barked trees, although the differences were not significant (df = 1, 28, *F*=2.72, p = 0.1103). Similarly, 3.4 ± 0.8 adults/cm emerged from trees with rough bark, but only 1.8 ± 0.5 adults/cm emerged from smooth barked trees, without significant difference (df = 1, 28, *F*=1.97, p = 0.17) (Figure 4B).

**Figure 5.**
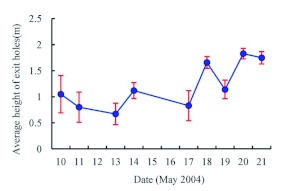
The height on the ash trunk where *Agrilus planipennis* adults emerged over time. High quality figures are available online.

The earliest exit holes, those that appeared on 10–13 May, appeared on the south or near south side of the trees. Adults emerging from the north side of the tree were the last to emerge, emerging around 20 May. Adults emerging from the bottom of the trunk emerged earlier than those emerging higher on the trunk (Figure 5). These findings suggest that the emergence of adults was related to the amount of time that the trunk was exposed to sunlight, as the young man-planted forests. The temperature of bark on the south side of the tree, which receives more sunlight, would be higher, and development of insects would be more rapid, resulting in earlier emergence. In the same way, the lower parts of host tree trunks are exposed to the sun for a longer time because they are not shaded by leaves, and the temperature would be higher than that of upper trunks; thus those insects in the lower parts of trunks would develop more quickly.

**Feeding and mating of adults.** The *A.* *planipennis* adults have to feed on ash leaves for nutrition after emergence. The leaves exhibited jagged edges after being consumed by adult *A. planipennis.* The *A. planipennis* adults chewed the edges of the leaves; they never bit the central leaf creating a hole in the leaf. Adults consumed some foliage every day, but the quantity of foliage consumed is unlikely to seriously damage trees.

*A. planipennis* adults favor sunlight but will drop to the ground and feign death or drop then fly if disturbed. They are active in the canopy under strong sunlight and high temperature conditions (> 25° C), frequently spreading their wings and flying short-distances from one tree to another. Adults hide in bark crevices or under leaves at night or when it rains. Adults seem to have good vision, and they will immediately flee when approached. There is some evidence suggesting that males find potential mates using visual cues (Lelito et al. 2007; Rodriguez-Saona et al. 2007).

**Figure 6.**
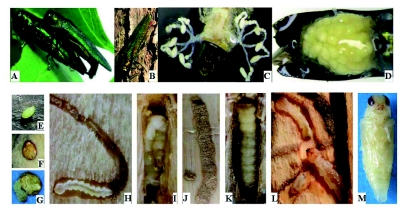
*Agrilus planipennis* life-stages and behaviors: A) Mating; B) Oviposition; C) The ovarioles of female adult prior to egg maturation; D) Eggs in ovarioles; E) Fresh egg; F) Mature egg; G) Hatching; H) Young larva in its gallery; I) Overwintering larva; J) Overwintering chamber (Pupal cell); K) Prepupa; L) Cannibalism of *A. planipennis* larva; M) Pupa. High quality figures are available online.

*A. planipennis* adults can mate multiple times based on the observations in laboratory, with the peak period of mating between 10:00 and 15:00. Mating was usually completed on the leaf surface (Figure 6A), but sometimes on the trunk. Generally, males were attracted to females sitting on leaves. On certain occasions, one female attracted two males simultaneously for mating. A coupling lasted 25 to 53 min, with an average of 39.5 min. Adults seldom flew when mating, except when disturbed.

**Oviposition and oviposition potential.** Adults begin to lay eggs after a pre-oviposition period of approximately 10 days for mating and egg maturation. Most oviposition occurred between 14:00 and 17:00 on sunny days. Females crawled slowly along the host tree trunk upwards or downwards to look for favorable oviposition sites. The female extended her long ovipositor frequently and quickly explored bark crevices. She laid an egg when she found an appropriate site, usually under bark flaps or in the vertical slits on the trunk (Figure 6B). Depositing an egg took a short time, usually about 2–5 s. Eggs were seldom exposed, making them very difficult to find. The sheltered eggs were presumably protected from bad weather, e.g. rainwater, and natural enemies. Eggs were laid on all cardinal directions of the tree, however females were observed searching for oviposition sites where the trunk was in the sun and presumably warmer, especially early in the season. Eggs were usually deposited individually, but sometimes several eggs were laid together. A maximum of seven eggs were observed in one location, but because the degree of development of those eggs was different, they may have been laid by more than one female, or by the same female at different times. *A. planipennis* eggs could be found on dead trees, and they also hatch naturally, but whether they can develop successfully is unknown. Adults have been observed to oviposit even on the surface of Petri dishes when reared in the laboratory.

The lifetime fecundity of an *A. planipennis* female in the field is difficult to quantify because eggs are usually laid in protected locations in bark crevices. The oviposition potential was determined through dissection of internal female reproductive organ under magnification. *A. planipennis* females have a pair of ovaries (Figure 6C), and each ovary consists of five telotrophic ovarioles. Each ovariole contains about 0–14 eggs and/or pre-eggs. The end of each ovariole dilates into a milk white sac. The mature eggs in ovarioles are straw yellow, with a diameter of 0.7–1 mm (Figure 6D), while immature eggs are cream-colored and small in size. The number of eggs observed in ovarioles varied considerably among females in different developmental stages. Immature females have few or no eggs in the ovarioles, and the hemocoel is filled with 60–130 white trophoroites (nurse cells) of various size, shape, and diameter (0.2–0.6 mm). Mature females contain abundant yellow, mature eggs and very few nurse cells in the haemocoel. Dissections of 21 females collected from the field throughout the season revealed an average of 32.62 ± 4.59 mature eggs (range 0–67) and 71.05 ± 7.12 immature eggs (range 0–140) per female adult.

### Eggs

Newly deposited eggs were ivory-white to jade-green (Fig. 6E), and then they became fulvous to brown (Figure 6F) after 3–4 days. Eggs were irregularly elliptical with a rough surface. They averaged 1.23 mm (range 1–1.4 mm) long, 0.96 mm (range 0.8–1.1 mm) wide, and 0.25 mm (range 0.2–0.3 mm) thick. Under ambient room temperatures averaging 22.9° C, the duration of the egg stage averaged 15.09 ± 0.20 days (range 12–19, *n* = 76). The eggs deposited in mid to late May hatched in 17–19 days at 18–23° C, while eggs laid in late June hatched after only 12–13 days at 24–26° C (Figure 6G).

### Biology of larvae

The larval stage was the longest of the life cycle, lasting from June to April of the following year, for a total of approximately 300 days. *A. planipennis* larva has four instars based on examinations of the length of the urogomphi, the width of the peristoma, and the width of the prothoracic plate (Wang et al. 2005).

**Morphology.** The body of the larva is prolate, translucent, and milk-colored. The brown head is mostly retracted into the prothorax and only the mouthparts are visible externally. The abdomen has 10 isoceles, trapezoidal segments without legs, and the 7th abdomenal segment is the widest. There is a pair of spiracles on mesothorax ventral-laterally and each of 1^st^-8^th^ abdomen segments dorsallaterally (Wang et al. 2005). The last segment has a pair of brown, pincer-shaped urogomphi. The late stage larvae reached an average length of 19.45 plusmn; 0.40 mm (range 10.7–37 mm, *n* = 143) and a width of 2.85 ± 0.06 mm (range 1.5–5.1 mm, *p* = 143). The sizes of full-grown larvae that entered the overwintering chamber were 13–22 mm in length and 3–4 mm in width. The larvae of *A. planipennis* look fragile and are relatively inactive.

**Feeding habits.** After hatching, the 1^st^ instar larvae chewed first through the chorion of the egg and then through the bark to enter the phloem. The inside of the egg was filled with frass after the larva hatched. The newly hatched larvae fed in the outer phloem and gradually bored into cambial region as development progressed. Few 1^st^ instar larvae reached the cambium. As they fed, *A. planipennis* larvae produced an S-shaped gallery that was formed in the cambial region (Figure 6H). There were some exceptions; a few larvae fed vertically and did not make S-shaped galleries, especially on young trees. Galleries were packed with fine frass that remained under the bark. Full-grown larvae entered into the xylem for building overwintering chambers. When *A. planipennis* larval density was very high on a tree, galleries crossed and connected with each other freely without clear direction.

**Figure 7.**
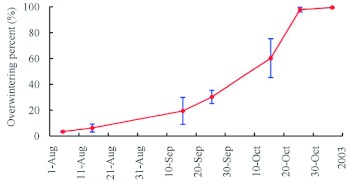
Percentage of *Agrilus planipennis* larvae in the overwintering stage over time (Mean value of every ten-day period in a month ± SE). High quality figures are available online.

Observation in the laboratory showed that *A. planipennis* larvae could enter into the xylem for feeding before overwintering. In the field, the larvae also entered the xylem in the early larval stages if the phloem and cambium had been entirely consumed because of a high larval density. An individual larva consumed a 35–41 cm long tunnel and about 9.14 cm2 in its lifetime. The phloem tissues became loose and slightly plump after attack. The bark of severely attacked trees would separate from the sapwood and fall off the tree as the transportation of water and nutrients was blocked by the feeding of many larvae, and finally the entire tree died.

**Overwintering.** Larvae entered the last instar stage (instar 4) after feeding for over 2 months. Full-grown larvae began to appear in late July. The percentage of overwintering larvae increased gradually, and most insects bored into sapwood for overwintering by late October (Figure 7). The full-grown larvae bored into the outer sapwood at the end of galleries to excavate an overwintering chamber of 13–28 mm in length, 3–7 mm in width, and 4–16 mm in depth under the bark, which was about 1–7 mm below the surface of the xylem sapwood. Exit holes were also constructed in the overwintering chamber at the end opposite the chamber entrance and were sealed with frass to prepare for adult emergence the following spring. The exit holes were 3–4.5 mm in length and 2–3 mm in width. The distance between the exit and entrance holes was 13–35 mm (Table 2). Finally, the fully grown larvae stopped feeding and overwintered, with their bodies shortening, broadening, and becoming J-shaped (Figure 6I). In general, the overwintering chambers were built vertically, and their direction was usually opposite to that of the former gallery produced in cambial region. For instance, if larva fed upwards in cambium, then the exit hole was usually located beneath the entrance hole (Figure 6J). When temperatures rose in the spring, the body of the larva straightened, the head extended towards exit hole, and the larva became broader and shorter (10–14 cm versus 16–22 cm in length). The insects began to enter the prepupal stage (Figure 6K), which that lasted 2–3 weeks. A few larvae (<1%) in Tianjin overwintered in their galleries (not in overwintering chambers) and continued development the following year, possibly taking two years to complete development. In Heilongjiang Province in northeastern China, *A. planipennis* is semivoltine, and both univoltine and semivoltine life-cycles occur in Changchun, Jilin Province (Wei et al. 2007). However, Liu et al. (2007) reported *A. planipennis* had a one-year life cycle in Changchun of Jilin Province, as well as in Liaoning Province.

### Winter dormancy

Overwintering *A. planipennis* larvae can tolerate very low temperatures in the winter. The supercooling point is between -26.38° C and -23.04° C, and the freezing point ranges from -21.72° C to -11.64° C in China (Wu et al. 2007). However, *A. planipennis* larvae seemed to overwinter in a dormant state and did not enter a true diapause. When overwintering *A. planipennis* larvae collected in November in ash branch segments were placed under room conditions (about 18–22° C), approximately 23% pupated in February to March 2004 and successfully emerged (Table 3). An even greater proportion of *A. planipennis* (around 88%) successfully emerged from log sections collected in September and caged in the laboratory under room conditions. When overwintering *A. planipennis* larvae collected in January were placed on an artificial diet in an incubator at 25 ± 1° C, and 60–85% RH, approximately 19% pupated and emerged one or two weeks later (Table 3). The mortalities of *A. planipennis* increased during the culture at room or incubator conditions from being easily infested by microorganisms or possibly from lack of moisture when reared by branch segments or artificial diets. *A. planipennis* adults emerged earlier in the year when reared under room or incubator conditions than in field or when they experienced the cold storage control treatment. Thus, the overwintering *A. planipennis* larvae can recover from dormancy and develop continuously when given appropriate conditions, especially higher temperatures. A period of low temperatures may not be necessary for completing the life cycle, perhaps indicating that ash trees in the Southern United States could support *A. planipennis* populations. However, it seems that some period of cold does speed up emergence.

**Table 2.**
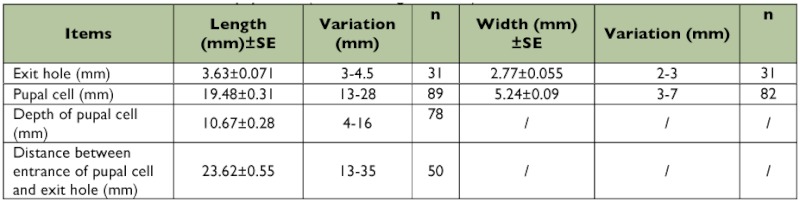
The sizes of exit holes and pupal cells (overwintering chambers)

**Ecological habits.** The results of field investigations demonstrated that *A. planipennis* larvae cannibalize when their tunnels cross (Figure 6L). Especially at higher population density, galleries crossed frequently, and *A. planipennis* larvae were more likely to encounter one another. Under these conditions, cannibalism was frequent, approximately 5%. Naturally, *A. planipennis* larval populations exhibit a negative binomial (aggregated) distribution due to the biology of the insect (Wang and Yang 2005).

### Pupae

*A. planipennis* pupa is exarate, rhombic (Figure 6M), 11–16 mm long and 3–5 mm wide. Initially the pupae were milk white, but the compound eyes changed to black after approximately 10 days. Over time, the entire body became brassy or golden green with a metallic luster. The average pupal stage duration was 20.06 ± 0.70 d (11–38, *n* = 62) at 18–20° C.

**Table 3.**
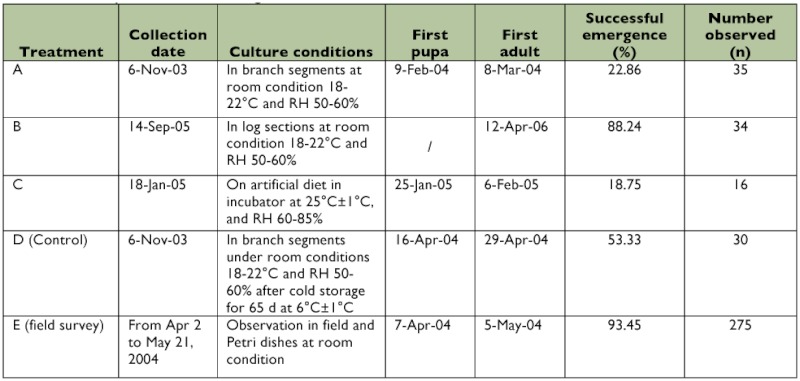
Development of overwintering EAB under different conditions

### Life cycle summary

*A. planipennis* was mostly univoltine in Tianjin, China (Table 4). The late stage larvae began to bore into xylem beginning in late July, and all insects entered the overwintering stage by late October to early November. They overwintered in pupal chambers in the outer sapwood as mature larvae. Larvae became prepupae from early through mid March of the following year. Pupation lasted from early April through mid May. Some new adults could be found in pupal cells by the end of April, but they did not exit. Adults first emerged in early May and fed on ash leaves after emergence. About one week later, the reproductive system of adults matured, and they then mated. Adults oviposited following mating and disappeared in late June. The egg stage was present from mid May through early July. The newly hatched young larvae could be found in early June in the phloem, and the larvae continued feeding until late October or early November before overwintering.

### Population density and damage status

**Figure 8.**
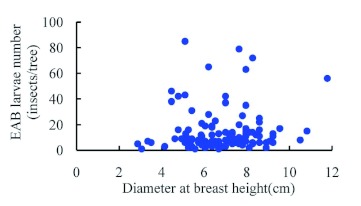
Relationship between *Agrilus planipennis* larval density and DBH of host tree trunks. High quality figures are available online.

While investigating the infestation of velvet ash trees from August to November 2003, few *A. planipennis* were found on trees of diameter less than 3 cm. *A. planipennis* most commonly attacked trees of 5–9 cm in DBH. Of course, these results are affected by the fact that the trees in the Guangang Forest Park were only 9–10 years old. *A. planipennis* density ranged from 0–20 larvae per tree, with only a few trees having infestations over 40 insects (Figure 8).

**Table 4.**
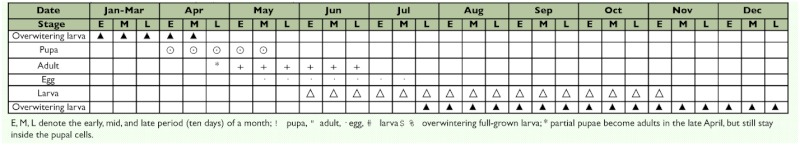
The life cycle of emerald ash borer (Tianjin.China, 2003–2004)

**Figure 9.**
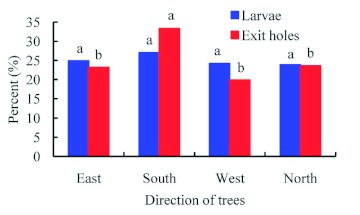
Distribution of *Agrilus planipennis* larvae and exit holes in different cardinal directions of host tree trunks. The different letters on top of bars in the same series show significant differences among different direction of trees at the α = 0.05 level. High quality figures are available online.

**Figure 10.**
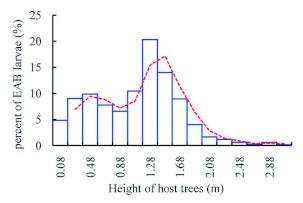
Distribution of *Agrilus planipennis* larvae at different heights on host tree trunks. High quality figures are available online.

**Figure 11.**
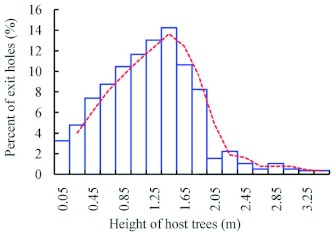
Distribution of *Agrilus planipennis* exit holes at different heights on host tree trunks. High quality figures are available online.

The distribution of *A. planipennis* larvae in the four cardinal directions of the trunks showed no significant difference (Figure 9) (χ^2^ = 2.4016, p = 0.4933, df = 3, *n* = 976), however, there were significantly more exit holes on the southern face of the tree trunk than on the other faces (χ^2^ = 18.6061, p = 0.0003, df = 3, *n* = 462). *A. planipennis* galleries were also found most often on the south-west side of the tree (Timms et al. 2006). *A. planipennis* larvae were located from 0–3.4 m in height on the trunk, with the majority found between 1 and 2 m, with a peak at 1.4 m (Figure 10). The distribution of exit holes was quite similar to that of larvae (Figure 11), peaking at 1.45 m.

### The geographical distribution of *A. planipennis* in China

According to references, *A. planipennis* was recorded in northeastern China (Heilongjiang, Jilin, Liaoning), Shandong, Inner Mongolia, and Hebei (Yu 1992), Tianjin (Zhang et al. 1995; Liu et al. 1996), and Taiwan (Jendek 1994). Ash trees were popular for use as ornamental trees, street trees, and in gardens and parks. Young trees or seedlings of ash trees were transplanted frequently, so *A.* *planipennis* spread to many other ash-growing regions. It was reported that *A. planipennis* could naturally disperse about 1.1 k per year (Liu et al. 1996), but transportation by humans was the major vector for the spread of *A. planipennis,* especially for long distance dispersal (BenDor et al. 2006; Muirhead et al. 2006). Hence, strict quarantine measures are necessary to avoid further spread.

The geographical distribution of *A. planipennis* was surveyed in China in late February to mid March 2004. *A. planipennis* was found in Benxi of Liaoning Province, Changchun of Jilin Province, Harbin and Shangzhi of Heilongjiang Province, Tangshan and Qinhuangdao of Hebei Province, but absent in Shandong Province and Inner Mongolia. In northeastern China, the host plant was *F. mandshurica* and *F. pennsylvanica*, and the infestation rates were low except in plantations on the campus of the Northeast Forestry University in Harbin, Heilongjiang Province. The densities of *A. planipennis* were also somewhat high on *F. velutina* in Tangshan of Hebei Province and in Tianjin City. In 2006, small-scale *A. planipennis* infestations were found in Beijing. Wei et al. (2004) reported *A. planipennis* might be found in Xinjiang Uygur Autonomous Region, but further confirmation is required.

### The natural control factors of *A. planipennis*

Several natural enemies were found attacking *A. planipennis* to varying degrees. The most important predators were woodpeckers, mainly the great spotted woodpecker, *Dendrocopos major*, and the grey-headed woodpecker, *Picus canus.* In addition, the common hoopoe *Upupa epops* was observed in ash forests and was possibly feeding on *A. planipennis* larvae. Results of a survey of 383 ash trees in the research plot showed that 14.74% (± 1.34%) of the trees showed attacks by woodpeckers, and the average predation on *A. planipennis* larvae on the attacked trees was 26.37%. The average percentage of larvae consumed by woodpeckers was approximately 3.89%. It was reported that woodpeckers were also a significant source of *A. planipennis* mortality in North America (Lindell et al. 2008).

At least 4 species of ants (*Plagiolepis manczshurica, P. alluaudi, Crematogaster terrorii,* and *C. egidyi*) were observed preying on *A. planipennis* larvae. They can enter into the galleries to find the larvae through bark crevices or holes created by woodpeckers.

The parasitoid, *Spathius agrili* (Yang et al. 2005), was found to be the dominant insect natural enemy of *A. planipennis* larvae in Tianjin. This braconid is a gregarious idiobiont ectoparasitoid. It has 3–-4 generations a year, can reach level of parasitism as high as 60%, and shows excellent potential for biological control (Wang 2005). An unidentified chalcidoid was also found parasitizing *A. planipennis* larvae, and a bethylid, *Sclerodermus pupariae* attacked *A. planipennis* pupae and prepupae (Wu et al. 2008; Yang and Yao 2009).

An ectoparasitic mite, *Pyemotes* sp., was found on the body surface of the larvae of *A. planipennis* and *Spathius agrili.* The mite was cream-colored with negative phototropism and a body size of about 0.375 × 0.17mm. The egg of the mite was also cream-colored, with a size of 0.125 × 0.075mm.

In northeastern China, a new egg parasitoid, *Oobius agrili,* was also described (Zhang et al. 2005), and a chalcidoid *Tetrastichus planipennisi* (Yang et al. 2006), and an ichneumonid, *Deuteroxorides orientalis,* were found parasitizing *A. planipennis* larvae and were collected from larval galleries. *T. planipennisi* was the locally predominant parasitoid (Liu et al. 2007) and is also thought to have potential as a biocontrol agent.

Some pathogens, such as *Beauverin bassiaua* and *Metarhizium anisopliae,* also attacked *A. planipennis* larvae (Liu and Bauer 2006). The infestation rate was approximately 10.62 ± 2.37% (range 1.63–21.76%) in Tianjin. Mortality of *A. planipennis* larvae from fungi increased notably following periods of rain.

## Suggestions for Integrated Control

The control of *A. planipennis* is difficult because most of its life cycle is spent concealed beneath the bark of host trees; it only occurs outside the bark in its adult stage for about one month. The results of this investigation point to a number of steps managers could take to reduce the impact of *A. planipennis* infestations. Although chemical insecticides were applied by the Guangang Forest Park each year, they were not effective in suppressing *A. planipennis* populations. In addition, chemical control strategies are expensive and harm non-target organisms, especially the predominant parasitoid *S. agrili* and *Sclerodermus pupariae*. Based on the results of this study, the following potential control strategies are suggested for the integrated management of *A. planipennis.*

### Proper silvicultural practices

In China, monoculture ash forests are more susceptible to attack by *A. planipennis.* Also, trees on the edge of a forest are more susceptible to attack. Planting forests with multiple species is recommended to imcrease the biodiversity and the natural enemy species in the forest, thereby enhancing the health and stability of the forest ecosystem.

### Timely forecasting and control

Because the larvae are difficult to control and adults must feed ash leaves for 10 days after emergence before mating and oviposition, the preoviposition period of the adult provides an excellent opportunity for control. This life stage could be controlled using methods such as selective insecticides (Nzokou et al. 2006; Petrice and Haack 2006), sexual attractants, or trapping (Francese et al. 2005; Lelito et al. 2008; de Groot et al. 2008).

### Releasing natural enemies for classical biological control

*Spathius agrili* is a promising biological control agent of *A. planipennis* and has been released in the United States. It has reproduced and overwintered in the field (JRG, personal observation), but it is too soon to evaluate effectiveness. Because some members of the genus *Sclerodermus* have been known to sting humans and do not tend to be host specific (Gordh and Mozcar 1990), this insect was not considered for release in the United States.

### Eliminating dead ash trees for reduction of insect innoculum

This study showed that the number of *A. planipennis* adults emerged from dead trees was significantly higher than from live trees. So, if conditions permit, e.g. in some parks or plantations with only small trees, surveys should be conducted regularly to identify seriously infested trees in the fields. Dead or dying trees should be felled and removed in time to destroy the insect inoculum of the next generation.

### Planting selected variety of ash tree with resistance

This study revealed that trees with smooth bark and without bark cracks were attacked less frequently. The parasitism rates by *S. agrili* were also higher on the trees with thin and smooth bark. Different species of ash trees also have different resistance against *A. planipennis,* e.g. *F. mandshurica* is more resistant to *A. planipennis* (Eyles et al. 2007), while the green ash *F. pennsylvanica* is most likely to be attacked by *A. planipennis* (Anulewicz et al. 2007). Thus culturing and planting resistant ash varieties are perhaps an effective strategy for the management of *A. planipennis*, especially if biological control agents establish in North America.

## **Abbreviations**

DBH: diameter at breast height
